# Charge and Spin Transport in Doped Rubrene Thin-Film Crystals

**DOI:** 10.1021/acsnano.6c02012

**Published:** 2026-03-16

**Authors:** Zichen Wang, Stephanie A. Buchholtz, Wooik Jang, Mike Hambsch, Stefan C. B. Mannsfeld, Xinglong Ren, Ian E. Jacobs, Henning Sirringhaus, Hans Kleemann

**Affiliations:** † Optoelectronics Group, Cavendish Laboratory, 2152 University of Cambridge, JJ Thomson Avenue, Cambridge CB3 0HE, United Kingdom; ‡ Dresden Integrated Center for Applied Physics and Photonic Materials (IAPP), Technische Universität Dresden, Nöthnitzer Straße 61, 01069 Dresden, Germany; § Center for Advancing Electronics Dresden and Faculty of Electrical and Computer Engineering, 9169 Dresden University of Technology, 01069 Dresden, Germany; ∥ Nano and Microelectronic System (NMES), Technische Universität Ilmenau, 98693 Ilmenau, Germany

**Keywords:** organic semiconductors, spin transport, spin relaxation processes, molecular doping, charge carrier transport, vertical spin valve

## Abstract

Organic semiconductors offer a long-spin coherence time and diffusion length due to the weak spin–orbit and hyperfine interactions in these materials. However, in commonly used lateral field-effect transistor structures, it is challenging to define device dimensions comparable to the spin diffusion length. On the other hand, vertical structures, offering smaller device dimensions, are facing issues due to the low carrier mobilities in the vertical dimension. Here, we investigate spin relaxation in rubrene thin films with a triclinic phase, which are doped with C_60_F_48_ by coevaporation. The doping provides an efficient way to generate charge carriers, and their high out-of-plane mobility should enhance long-spin diffusion. Using electron-spin resonance, we show that the spin relaxation is governed by the interaction with the dopant counterions and estimate the spin diffusion length to be ∼200 nm. This is comparable to the film thickness, which should make such doped rubrene films an attractive system for spintronic device applications.

## Graphical abstract

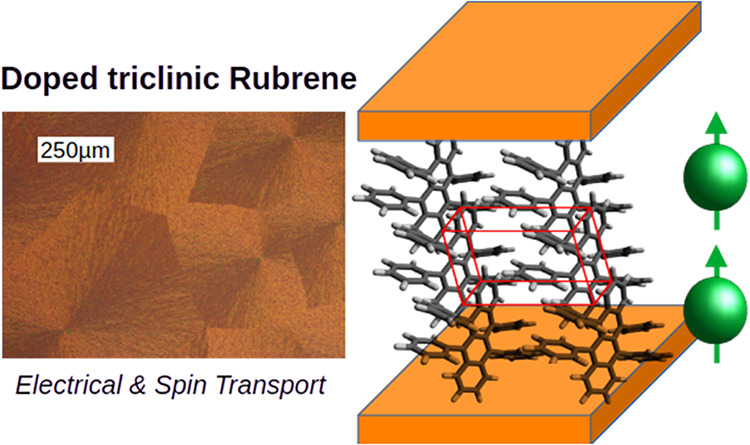

## 1 Introduction

Spin is a phenomenon that is inherently linked to quantum mechanics. Hence, the state of electrons in a crystalline semiconductor is described not only by energy and momentum but also by the spin quantum number, which adds an important degree of freedom to the electronic system and provides the basis for spintronics and quantum computing, which are expected to revolutionize the field of microelectronics. However, it remains a big challenge to exploit the spin degree of freedom in electronic devices due to the short spin diffusion length/fast spin relaxation time in conventional inorganic semiconductors, such as GaAs, due to relatively strong spin–orbit coupling and efficient spin scattering.^1^

Organic semiconductor materials have gained increasing interest in recent years, due to their long-spin lifetime and unique electrical responses to light and external stress, which offer new possibilities for spin manipulation.^2–4^ Since these semiconductor materials are mainly composed of light elements such as carbon or hydrogen, spin–orbit coupling and hyperfine interactions are weak, which is a prerequisite for long spin coherence. Using low-defect single-crystalline organic thin-film crystals, long spin lifetimes of ∼10 μs have been demonstrated at low temperatures^5^ with a corresponding diffusion length in the range of 1 μm, which is comparable to what has been observed in transition metal dichalcogenides with topologically protected spin states.^6^ However, also for amorphous polymer films, the spin lifetime exceeds several hundred nanoseconds and exhibits a very similar temperature dependence to molecular crystals.^7^ This has been interpreted as evidence that in both polymers and molecular crystals, spin relaxation is influenced by the strong coupling between spin/charge motion and the structural, vibrational dynamics of the molecular environment.^8^ In general, different spin relaxation mechanisms, such as Elliot–Yafet-like relaxation due to spatial scattering,^7^ relaxation due to motional narrowing,^7,9,10^ and conventional Elliot–Yafet momentum scattering (with the presence of heavy atoms),^11^ have been observed.

Despite the substantial progress about the understanding of spin transport inorganic semiconductors in the last couple of years, the spin diffusion length λ, which has been observed to be typically <1 μm, is too short to exploit the spin degree of freedom in lateral field-effect transistor structures, which is why vertical spin devices with a thickness *t* similar to the spin diffusion length are preferable (device thickness *t* ≈ λ). Unfortunately, for the vast majority of small molecule semiconductors as well as conjugated polymers, the vertical direction is the out-of-plane direction of transport, which exhibits a low charge carrier mobility and reduced spin diffusion length. Furthermore, probing spin transport in the vertical direction using the giant-magnetoresistance effect (spin valve)^12^ or spin-pumping experiments^13^ has been demonstrated to suffer from artifacts,^14^ which makes it challenging to investigate the link between charge carrier and spin transport in these vertical devices. Electron-spin-resonance spectroscopy (ESR) provides a reliable method for probing spin relaxation and spin transport; however, it has rarely been employed for vertical spin devices based on molecular semiconductors.^15^ The reason for that lies in the fact that in field-induced ESR based on field-effect transistor architectures, the required spin signal (>10^12^–10^13^ spins) can be induced simply by applying a sufficiently large gate voltage. In vertical thin-film devices, though it is more challenging to reach this number of charges/spins, this would require a large concentration of bulk dopants (>10^18^ cm^–3^, e.g., assuming an active area for the ESR or 5 mm^2^ and a thickness of the layer of 1 μm), which might deteriorate the microstructure and charge transport.

An organic semiconductor material system, which is, however, promising for vertical spin transport, is rubrene (Rub, see Figure 1a). It is known to be among the organic semiconductor materials with the highest charge carrier mobility, and most interestingly, it may crystallize in multiple thin-film polymorphs, preferring either lateral or vertical transport with respect to the substrate plane. In particular, the triclinic phase (see Figure 1a,b) is ideal for vertical transport^16^ since the overlap integral of the frontier orbitals is greatest in the vertical direction. Thus, if such rubrene thin-film crystals can be efficiently chemically doped, it would be an ideal model system to study spin-transport in a high-mobility organic semiconductor material using ESR in a vertical device structure, possibly allowing for a spin diffusion length comparable to the device thickness (*t* ≈ λ).

**Figure 1:**
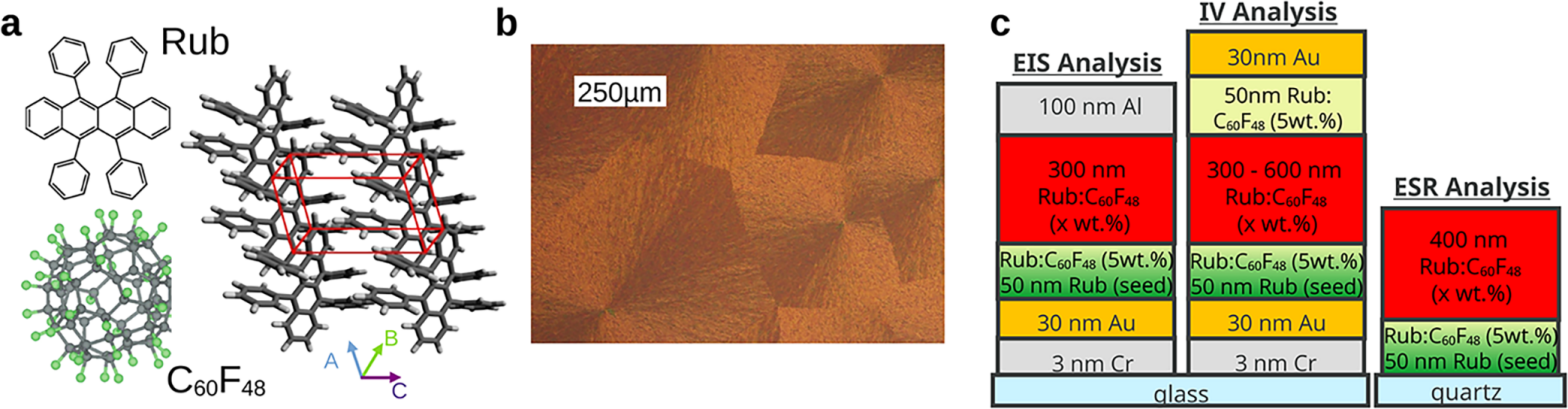
(a) Molecular structure of rubrene (Rub), C_60_F_48_, and an illustration of the rubrene molecular lattice in the triclinic phase. (b) Optical micrograph (measured in reflection using a polarization filter) of a doped rubrene (5 wt % of C_60_F_48_) thin-film layer showing the typical structure of the triclinic spherulites. (c) Illustration of the device stacks used for electrochemical impedance spectroscopy (EIS) (Schottky contact between Rub and aluminum, left), symmetric p^+^-p-p^+^ devices to measure the vertical electrical conductivity via IV analysis, and the stack for the electron-spin resonance measurements (ESR). For the devices used for EIS and IV analyses, the seed layer (green) is composed of 50 nm of undoped Rub (crystallized via rapid heating) and 50 nm of doped Rub (with 5 wt % of C_60_F_48_) deposited on top of the crystallized seed (see Section 4 for details).

Here, we show that rubrene thin films in the triclinic phase doped by the strong p-type molecular dopant C_60_F_48_ are indeed promising candidates for such vertical organic spintronic devices, providing both a sufficient number of spin carriers and long-spin diffusion lengths comparable to the device thickness. By physical vapor codeposition of rubrene and C_60_F_48_ on a precrystallized rubrene seed layer, we can achieve efficient doping of the rubrene films, introducing up to ∼10^19^ cm^–3^ holes as charge/spin carriers without any noticeable change to the crystal structure. We conducted a systematic study of charge transport and spin dynamics and found evidence for a long-spin diffusion length of >200 nm in the vertical direction. For this, we first evaluated the carrier concentration under relatively low doping levels by electrochemical impedance spectroscopy using Mott–Schottky (MS) analysis of the depletion capacitance. By analyzing the temperature dependence of this capacitance, we observed an increase in activation energy of the doping process with the increased doping levels, which is attributed to the reduction in chemical potential with the increased number of dopants/carriers. Moreover, using DC conductivity measurements, we estimated the charge mobility to be larger than 1 cm^2^/(Vs) in the vertical direction, independent of doping levels. The spin dynamics of the induced carriers were studied by electron-spin resonance. We find evidence that spin relaxation times of charge carriers in doped rubrene films are much shorter than those for field-induced charges and are determined by interactions with the dopant counterions. However, the spin relaxation times are still long enough to enable spin diffusion lengths comparable to the thicknesses of the films.

## 2 Results and Discussion

The rubrene thin-film crystals are created by transforming an initially amorphous layer of rubrene (named here as seed) by rapid heating into a polycrystalline layer. This approach, which was originally proposed by Park et al.,^17^ enables us to select the polymorph of the thin-film crystals by the temperature of the heating process. Furthermore, once the crystalline seed layer is formed, additional layers of rubrene deposited afterward adapt to the stacking motif of the seed in an epitaxial growth mode. As previously shown, even doping of the rubrene crystals by codeposition is feasible, enabling complex device structures such as pin-diodes, bipolar transistors, or organic light-emitting diodes.^18–20^ In this work, we focus on the triclinic polymorph of rubrene (130 °C for rapid heating; details of the fabrication processes are given in Section 4) as it is more suitable for vertical transport than the orthorhombic phase due to the denser packing along the *c*-axis. The highest occupied molecular orbital (HOMO) of triclinic Rub is measured at −5.43 eV w.r.t. vacuum with ultraviolet photoelectron spectroscopy (see Section 1 of the Supporting Information), which matches with other reported values.^21^ We selected, for our study, the p-type dopant C_60_F_48_ as its high electron affinity (−5.0 to −5.5 eV)^22^ is expected to provide efficient doping. Figure 1b shows a representative micrograph of the doped Rub films in the triclinic spherulite phase. As discussed below in more detail, but also shown in ref 29, the codeposition of the dopants does not disturb the RUB crystal formation for dopant concentration <5 wt %. This surprising finding is supported by X-ray diffraction experiment, polarized light microscopy, and scanning force microscopy. Furthermore, we do not indicate a preferred accumulation of dopants at RUB grain boundaries. Hence, we assume here that the distribution of dopants is uniform.

### 2.1 Electrochemical Impedance Spectroscopy and DC Conductivity Analysis

Using the epitaxial growth method described above, we fabricate functional electronic devices for electrochemical impedance spectroscopy (EIS) and DC conductivity (IV) analyses. We utilize a Schottky junction appearing between the doped Rub layer and a top aluminum electrode (see Figure 1c) to study the formation of a charge depletion zone (CDZ) using Mott–Schottky (MS) analysis.^23,24^ If the doping concentration is not too high (<5 wt %), the Schottky diodes show a rectification in the current–voltage curve (see Section 2 of the Supporting Information; Figure S2), which is sufficient for analyzing the CDZ by EIS. Most importantly, the low-frequency impedance (<100 kHz) shows a phase reaching almost −90° (see Figure S4), meaning that a capacitance can be extracted under the assumption of an RC parallel circuit. This capacitance is assigned to the formation of a charge depletion zone within the abrupt-junction approximation. As further shown in Figure 2a, the capacitance plateau is obtained even for various temperatures, enabling us to determine the activation energy^24^ of the doping process as discussed in the following (raw data of the EIS analysis for different dopant concentrations and temperatures are given in Section 2 of the Supporting Information; Figures S3 and S4, and additional comments on the analysis). Note that the capacitance vs frequency curve shows a weak frequency-dependent behavior, which is often assigned to the presence of trap states. However, we see this weak frequency-dependent behavior to be neither voltage nor temperature-dependent, a behavior that would be expected, though when changing the filling of such trap states. We hypothesize that this weak frequency-dependent behavior is instead due to the high electrical conductivity of the doped layers, leading to lateral spreading of the charge carriers in the vertical device structure as the doped layer of rubrene is unpatterned.^25^ This hypothesis is supported by the finding that the frequency dependence is the weakest for lightly doped samples (see Figure S4). Furthermore, as the frequency-dependent capacitance contribution is almost voltage independent (C–f curves are just offset for different voltages), it does not influence the analysis of the doping process using the Mott–Schottky method. Finally, we emphasize that the increase of capacitance at low frequency (<100 Hz, in particular at high temperature) is caused by a parallel resistor (not taken into account when computing the capacitance from the raw data), representing leakage current over the Schottky barrier (due to the doping). For the Mott–Schottky analysis, though we only use impedance data from the capacitance plateau, the parasitic resistance can be neglected.

**Figure 2:**
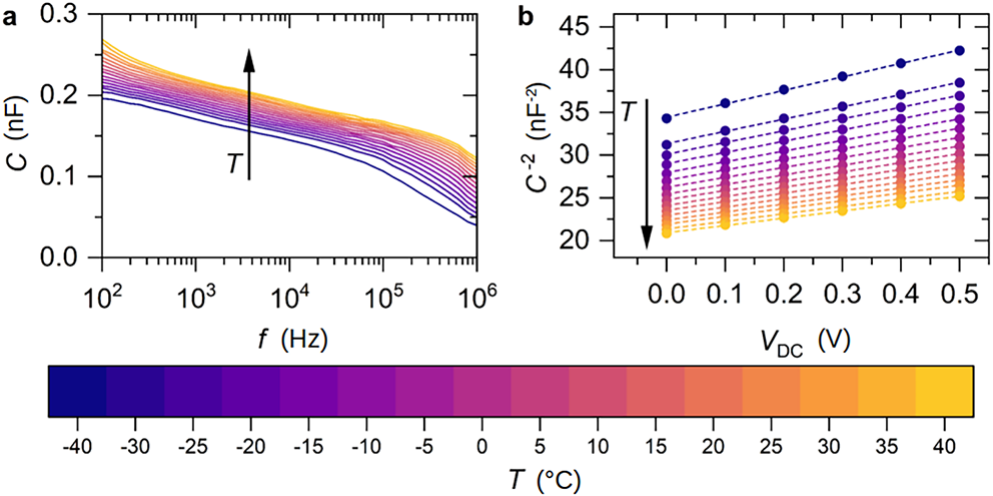
(a) Capacitance over frequency and (b) Mott–Schottky plot with linear fits for Rub:C_60_F_48_ (0.5 wt %) in a Schottky diode with a layer thickness of 300 nm and an active area of 0.25 mm^2^ for various temperatures between −40 and 40 °C. High potential is applied at the top electrode, which is the aluminum contact and therefore the expected Schottky contact.

Having confirmed the suitability of the capacitance–frequency behavior for different DC voltages, we set the small-signal AC signal to a fixed frequency of 1 kHz and analyze the widening of the CDZ when the junction is biased in reverse direction using a DC voltage of up to 0.5 V. Figure 2b shows the 1/*C* ^2^ vs voltage plot for different device temperatures for a dopant concentration of 0.5 wt %. From the absolute value of the capacitance, we can estimate the width of the CDZ, while from the slope, we can derive the density of ionised acceptor states *N*_A_^–^ using the relation

1 $$N_{A}^{-}=\frac{2}{\epsilon \epsilon_{0}eA^{2}}{\left(\frac{d\frac{1}{C^{2}}}{dV}\right)}^{-1}$$

with *e* being the elementary charge, *A* being the active area defined by the overlap of the metal electrodes, and *V* being the applied DC offset voltage for the impedance analysis. ε and ε_0_ are the relative dielectric constant of Rub and the vacuum permittivity, respectively. Ignoring the weak frequency-dependent capacitance at low frequencies, the approach described above implies that we assume a uniform CDZ with abrupt boundaries. This analysis is repeated for samples with different dopant concentrations (0.5, 1, 1.5, and 3 wt %). The results of this analysis (density of ionised acceptors *N*_A_^–^ and activation energy of doping *E*_act_) are summarized in Table 1. As expected, we find that the concentration of ionized acceptors *N*_A_^–^ increases monotonically with doping concentration. The comparatively low density of ionised dopants at 0.5 wt % of doping is presumably due to the presence of tail states in the energy structure of the semiconductor.

**Table 1:** Density of Dopant Molecules *N*_C60F48_ (Calculated from Doping Levels in wt %) and Ionised Acceptors *N*_A_^–^ (Calculated from MS Measurements) at Room Temperature and Activation Energies of Doping *E*_act_ of Rub:C_60_F_48_ (Calculated from Temperature Dependence of *N*_A_^–^ in MS Measurements) for Various Dopant Concentrations^a^

*c* (wt %)	*N*_C60F48_ (10^18^ cm^–3^)	*N*_A,20 °C_^–^ (10^18^ cm^–3^)	*E*_act_ (meV)
0.5	2.491	0.88	49
1.0	4.982	3.79	10
1.5	7.472	5.38	8
3.0	14.944	9.48	52

a Note that the values presented in this table for *N*_A_^–^, 20 °C, and *E*_act_ are mean values determined from 4 samples for each dopant concentration, which were fabricated simultaneously. The systematic error of the measurement is <5%. However, the main contribution to the uncertainty of the measurement comes from the value of the dopant concentration (in wt %). Due to the calibration of the quartz crystal microbalances and the low dopant concentration, the relative uncertainty of the dopant concentration is 20.30%.

Our measurements show that the activation energy of the doping process is ∼10 meV for the 1 and 1.5 wt % samples, while it increases to 50 meV for the lowest and highest dopant concentrations. We hypothesize that the higher activation energy for the lowest dopant concentration is due to the filling of tail states.^26,27^ The increase in the activation energy for 3 wt % is, however, surprising. As we discuss later, this finding cannot be connected to a change in the crystal structure due to the higher dopant load; instead, we speculate that the increased activation energy is because the chemical potential of the doped rubrene crystal approaches the acceptor level, leading to incomplete ionization due to the extended density of states or an interaction of dopants.^27,28^ This is different from the situation at lower dopant concentrations, where the chemical potential might still be between the acceptor level and the midgap level.^26^ In any case, the values obtained for the density of ionized dopants (*N*_A_^–^) can be seen as an upper limit for the density of mobile charge carriers, and the linear relation between the dopant density (except for the lowest dopant density of 0.5 wt %) and the measured density of ionized acceptor density provides evidence for the effectiveness of the doping process. We would like to emphasize that due to the high molecular mass of the dopant (1632 g/mol) compared to the host (532 g/mol), the molar ratio of dopant and host is ∼3× smaller than the weight ratio. That means, e.g., 1 wt % of doping, the molar ratio is only 0.33 mol %. However, the dopant density is still much lower than the density of host molecules, and hence, we assume that the dopants are not interacting and are homogeneously distributed.

In the following, we analyze the charge carrier transport in these triclinic films as a function of the doping concentration to see whether the charge carrier mobility is influenced by the dopant density. Due to the supposedly high charge carrier density (∼10^18^ cm^–3^) and the preferred out-of-plane transport, methods such as Hall-effect measurements are not applicable. Instead, we analyze the current–voltage curves in p^+^-i-p^+^ and p^+^-p-p^+^ devices (Figure 1c) to determine the electrical conductivity and mobility. In Figure 3, we show the current–voltage characteristics of an intrinsic layer of triclinic rubrene (720 nm) sandwiched between two thin, highly doped triclinic rubrene layers for charge carrier injection (40 nm each). While below 1 V, we obtain a linear current–voltage relation, and the IV curve shows a slope between 1.9 and 2.1 in the voltage range above 1 V, indicating a space charge limited current (SCLC). In agreement with our previous study,^16^ we extract a charge carrier mobility of μ_SCLC_ = 1.89 ± 0.12 m^2^/(V·s) using the Mott–Gurney law (average over 13 devices).

**Figure 3:**
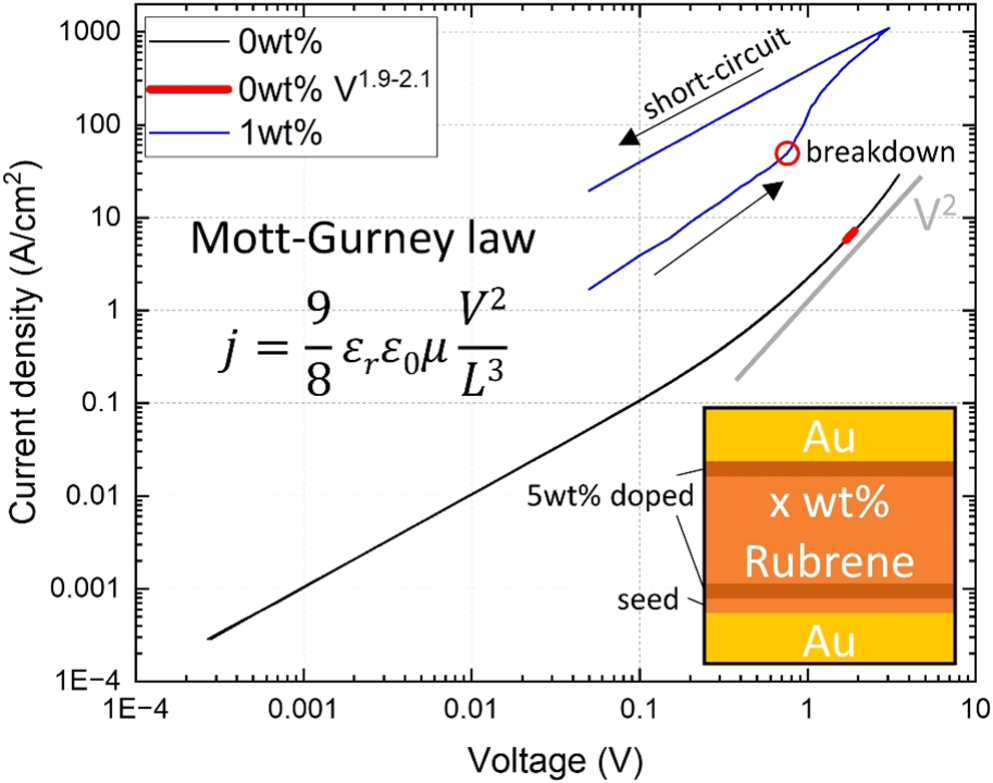
*I*–*V* characteristics of metal–semiconductor–metal devices of intrinsic and 1 wt % triclinic rubrene doped with p-dopant C_60_F_48_ sandwiched between 5 wt % doped injection layers grown on 40 nm triclinic rubrene seed. Hole mobility of the intrinsic device is determined by the Mott–Gurney law in the SCLC regime, strictly between the power law of 1.9 and 2.1 with ϵ_r_ = 2.62. Averaged mobility value of a total of 13 devices μ_SCLC_ = 1.89 ± 0.12 cm^2^/(V·s) for the undoped devices. The averaged linear conductivity of the doped devices is 0.1 ± 0.05 S/cm (12 devices). Device area: intrinsic 75 μm × 75 μm, 1 wt % doped 125 μm × 100 μm. Total semiconductor thickness: intrinsic 720 nm, 1 wt % doped 680 nm.

Adding dopants (C_60_F_48_) to the middle layer significantly increases the conductivity of the layer stack (see Figure 3). Even for a low dopant concentration of 0.5 or 1 wt %, we are not able to obtain a clear SCLC regime from these devices. We observe a linear current–voltage increase above 1 V. However, at current densities >100 A/cm^2^, the static current voltage measurement becomes unreliable. In fact, the majority of devices show irreversible damage when they exceed these current densities. For this reason, we define a “breakdown” point of 100 A/cm^2^, up to which it is safe to operate these devices and up to which it is reliable to extract conductivity values. From the derivative of the current–voltage curve, we obtain the maximum conductivity of the p^+^-p-p^+^ devices of 0.1 ± 0.05 S/cm in the linear regime. Taking μ_SCLC_ from the intrinsic layer and the density of dopants for 1 wt % of C_60_F_48_ from Table 1, we would expect an ohmic conductivity of 0.3 ± 0.06 S/cm, which is only slightly above the measured value. Based on the carrier concentrations and the measured conductivities, we can conclude that the charge carrier mobility in the doped layers is on the order of 1 cm^2^/(Vs). Note that, compared to the EIS analysis of the dopant activation resulting in an activation energy of tenths of meV, the thermal activation of electrical transport (doped and undoped films) is usually higher (100–150 meV),^29^ suggesting an additional thermal activation of the charge carrier mobility.

Furthermore, we would like to note that our comparison of the transport in the intrinsic and doped rubrene provides only a very indirect estimate. Moreover, the estimate relies on several key assumptions. First, the mobility of mobile charges in the doped and intrinsic rubrene layer is the same, implying that adding dopants does not lead to effects such as ionized impurity scattering or dopant-induced structural changes of the crystal structure. As discussed below, we believe that both assumptions are validated for low to moderate dopant concentrations (<3 wt %). Second, we assume that the density of ionized dopants is equal to the density of free charges and that this density is uniform across the layer. This assumption might be violated in the presence of deep trap states. However, the low activation energy of dopant ionization and the low-frequency plateau of the capacitance speak against the presence of such deep states in our rubrene films. Overall, the comparison of the calculated and measured conductivity for the doped film shows a good agreement and hence supports our reasoning. The fact that the measured conductivity is slightly lower than the calculated one, implies that either the charge carrier mobility in the doped films is slightly lower than in the undoped films or the density of free charges is slightly lower than the density of ionized dopants.

### 2.2 Structural Analysis of Doped Films

The interpretation of the IV analysis is supported by our X-ray diffraction analysis, proving that the crystalline structure of the films is maintained even for high dopant concentrations of up to 5 wt % (see Section 3 of the Supporting Information; Figures S5–S7 and ref 20). However, it is an open question whether these doped crystals remain stable even if the crystal undergoes a temperature-induced phase transition, which is quite common for rubrene. In particular, in preparation for the spin transport study, we aim to understand the implications of the previously reported low temperature phase transition of the rubrene crystals at ∼200 K,^30^ which has also been reported to be the reason for a sudden change in the temperature dependence of the charge carrier mobility (mobility crossover^30^).

The 2D grazing-incidence wide-angle X-ray diffraction (GIWAXS) images in Section 3 of the Supporting Information, Figures S5 and S6, show the diffraction pattern for rubrene with doping concentrations of 1, 3, and 5 wt % of C_60_F_48_ measured at 295 K. It can be seen that there is no significant change in the scattering signals. The observed signals can be described well by the previously reported triclinic unit cell (*a* = 7.02 Å, *b* = 8.54 Å, *c* = 11.95 Å, α = 93.04°, β = 105.58°, and γ = 96.28°) except for the ring at *Q* = 0.48 Å^–1^.^31^ We attributed this ring to small grains of the orthorhombic phase of rubrene, which could be present in the film as a minority phase besides the triclinic phase. The fitted unit cell parameters for the different C_60_F_48_ concentrations are summarized in Section 3 of the Supporting Information, Table S1, and it can be seen that the values are very close to the ones reported in the literature. We then investigate the evolution of the crystal structure of the film with 1 wt % of C_60_F_48_ when cooling it down. The 2D GIWAXS images are shown in Section 3 of the Supporting Information, Figure S7, for the temperature range of 135–295 K, and it can be seen that there are no major changes to the crystal structure. For a more detailed look, we also plotted the in-plane (Section 3 of the Supporting Information; Figure S7a) and out-of-plane (Section 3 of the Supporting Information; Figure S7b) intensity profiles, where we can see small peak shifts for some of the characteristic signals. To gain a better understanding of what these shifts represent, we calculate the unit cells at the various temperatures (Section 3 of the Supporting Information; Table S2) based on the observed diffraction signals, assuming that the angles are not changing and we only have an expansion or contraction of the lattice vectors. In Section 3 of the Supporting Information, Figure S7c, we show the temperature dependence of the lattice vectors and the unit cell volume, and we observe a clear linear trend toward reduced vector lengths and therefore smaller unit cell volumes with decreasing temperature. The largest reduction in length was observed for the *a*-axis with 2.8% when cooled from room temperature (295 K) to 135 K with a large step in the *a*-axis length vs temperature at 200 K. The overall volume of the unit cell was reduced by around 4.5% over the same temperature range. Additionally, we calculated and plotted the coherence length from the signals of the (001) and (010) planes at the various measured temperatures (Section 3 of the Supporting Information; Figure S7d). The coherence length can be considered the lower limit for the domain size, with actual grains potentially being bigger (as often seen in AFM images) due to other factors, like inhomogeneous strain and lattice imperfections.^32^ It can be seen that the coherence length remains mostly constant over the whole temperature range measured, and the average coherence lengths are around 29 and 27 nm for the (001) and (010) planes, respectively. Overall, we argue that the temperature change does not impose a significant change to the rubrene crystal structures. The abrupt change in the *a*-axis lattice parameter at 200 K seen in the doped films has been reported on undoped rubrene single crystals in the orthorhombic phase,^30^ where the expansion of the crystallographic axis is proven to come with a reduction in the electronic coupling and hence hole conduction by means of density functional theory (DFT) simulations.

### 2.3 Electron-Spin Resonance Spectroscopy

To characterize the spin relaxation processes in doped rubrene samples, we have used ESR and took a series of power-dependent cw-ESR scans at various temperatures from 5 to 290 K. An example of ESR spectra (with varied MW power) is shown in Figure 4a. The ESR peak centered at 3342.5 G can be attributed to the mobile carriers on rubrene induced by doping, as it appears at the same position and *g*-factor *g*_h_ = 2.0023 as field-induced charges in rubrene.^10^ The accompanying signal from the C_60_F_48_ anions generated in the doping process, despite their open-shell, spin-1/2 electron configuration, is not likely to overlap with the measured peak of the rubrene mobile carriers. According to the reports from refs 33,34 , the spins from most C_60_ anions have a *g*-factor close to *g*_h_ = 2.0000, and it is not likely that the fluorine substitution in C_60_F_48_ shifts the *g*-factor by a large amount to accidentally overlap with the rubrene signal^35,36^ (details about other possible origins of the ESR signals are included in Section 4 of the Supporting Information). At each temperature, we extract the susceptibility χ, spin–lattice relaxation time *T*_1_, and spin–spin relaxation time *T*_2_ by fitting to the power-dependent ESR spectra, respectively (more details about the χ, *T*_1_, and *T*_2_ extraction are shown in Section 5 of the Supporting Information). We compared the fitting residuals (defined as $R=\sqrt{\frac{1}{N-2}{\left(s-s_{\mathrm{fit}}\right)}^{2}}$ , with the number of point *N*, experimental and fitted values at each point sand *s*_fit_, respectively) with Gaussian, Lorentzian, and Voigt fit function to describe the experimental spectra at all temperatures and found that a Voigt function is preferable for all temperatures except room temperature, where a Lorentzian function yields a comparable fitting result (Section 5 of the Supporting Information; Figure S11).

**Figure 4:**
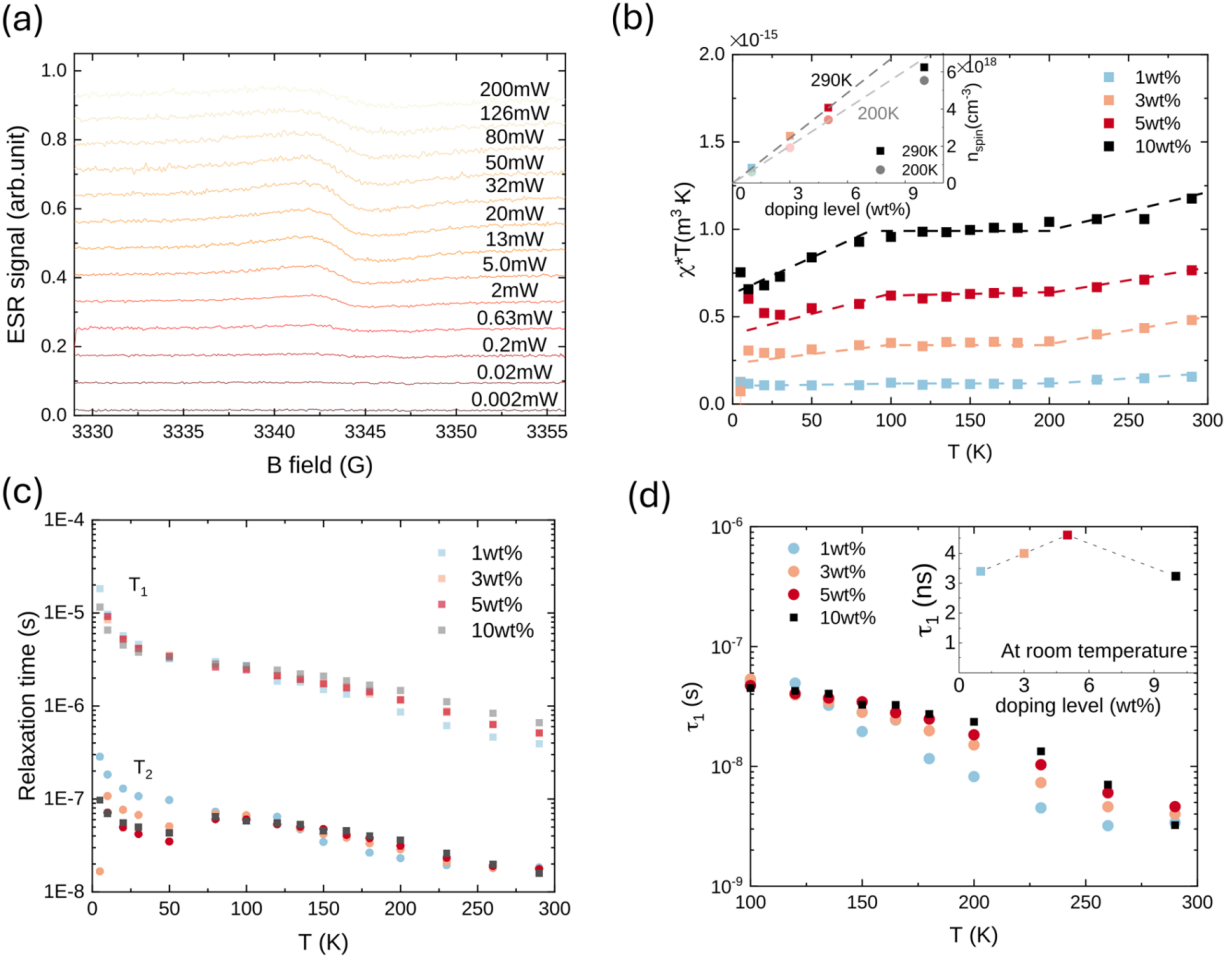
Power saturation spectra of cw-EPR of C_60_F_48_-doped rubrene samples: (a) example cw-EPR spectra measured with 3 wt % doped rubrene at 290 K; (b) product of susceptibility and temperature plot against temperature, inset: spin concentration extracted from ESR susceptibilities at various doping levels assuming the susceptibility is dominated by the Curie contribution (*n*_spin_ ∝ χ, based on eq 2), and the linear dashed lines are guides to the eye; (c) spin–lattice and spin–spin relaxation times at various temperatures; and (d) extracted characteristic interaction time τ_1_ extracted from eq 4 at various temperatures, inset: the characteristic interaction time τ_1_ at room temperature with various doping levels.

We plot the temperature-dependent spin susceptibility χ multiplied by temperature χ·*T* as a function of temperature, to distinguish contributions from the Curie susceptibility of localized carriers χ_C_ ∝ *C*/*T*, and a temperature-independent Pauli susceptibility χ_P_ from band-like mobile carriers (Figure 4b). The latter could potentially arise if the system were degenerately doped, i.e., if the Fermi level came to lie within the density of states of the HOMO-derived band. We consider this unlikely at doping levels below 10^19^/cm^3^, because simulations predict that at such low doping levels, the Fermi level is expected to remain >50 meV away from the band edge.^10^ Indeed, for doping levels of 1–5 wt %, the χ·*T* plots are approximately independent of temperature between 100 and 200 K, as expected for a predominant Curie contribution. We find that in this regime, the susceptibility increases approximately linearly with doping concentration (Figure 4b inset), providing further evidence that the susceptibility reflects localized carriers. Below 100 K, we observe a small decrease in χ·*T*. This could be explained by a freezing out of the dopant activation when the thermal energy becomes comparable to the dopant activation energy discussed above, around 100 K (*k*_B_ *T* ≃ *E*_A_ ∼ 10 meV). Above 200 K, we detect a clear increase of χ·*T* with increasing temperature; the 200 K transition temperature coincides with the phase transition of triclinic rubrene,^30^ which results in the reduction in lattice constants for the carriers in the range 200–290 K.^37,38^ At the highest doping level of 10 wt %, this increase becomes more pronounced. This may be correlated with the high dopant activation energy observed for this doping concentration and may suggest that the increase of χ·*T*, which is generally observed in all of the doped samples when approaching room temperature, may be a consequence of a more complete activation of all the dopants in the sample. It is also possible that in the 10 wt % sample, the rise of χ·*T* is due to a small Pauli contribution, which can be detected when the charge density approaches the metallic regime.^30,37,38^ Even so, the approximate proportionality of the susceptibility with doping concentration is clear evidence that the susceptibility is dominated by the Curie contribution from individual spins.

However, there are some discrepancies in the number of spins extracted from the MS data and the spin density *n*_spin_ estimated from the measured Curie susceptibility at temperature *T* (with Bohr magneton μ_B_, Boltzmann constant *k*_B_, *g*-factor of holes *g*_h_ = 2.0023, total angular momentum number *J* = 1/2, and volume of the sample *V*)­

2 $$\chi_{C}=\frac{\mu_{0}g_{h}^{2}\mu_{B}^{2}}{3k_{B}T}\cdot n_{\mathrm{spin}}V\cdot J\left(J+1\right)$$

The latter are by a factor of 3–4 smaller in the range of doping concentrations, where both techniques can be compared (inset of Figure 4b). This may be because some carriers may form spinless species, such as bipolarons. This effect should be more significant at high doping levels and may be the reason why the susceptibility at 10 wt % doping falls below the linear extrapolation from the lower doping levels^10,39^ (for a detailed discussion, see Section 6 of the Supporting Information). Finally, it is also worth pointing out that the temperature dependence of χ·*T* is not as strong as would be expected from the activation energy for dopant activation extracted from the MS and also does not drop to zero but remains at a constant value at cryogenic temperatures, where the motion of the induced carriers away from their counterions is frozen out,^40^ as evidenced by the temperature activated conductivity. Both observations suggest that the ESR susceptibility measurement is not able to distinguish between localized carriers, which are Coulombically bound to their counterions, and delocalized carriers, which are contributing to the high vertical conductivity.

We also extracted the spin relaxation times *T*_1_ and *T*_2_ as a function of temperature (compare to refs 5,7 ). The spin–spin relaxation lifetime *T*_2_ characterizes the decoherence for spin-precession in a plane perpendicular to the magnetic field and is determined from the line width of the ESR spectra, *T*_2_ ≃ 2/γ_e_Δ*B*_0_ (γ_e_—gyromagnetic ratio of the electron). The spin–lattice relaxation time *T*_1_, on the other hand, is the time for a spin excited to the upper Zeeman energy level to exchange energy with the lattice and return to the ground state; it is determined from the microwave power dependence of the ESR spectra. With increasing temperature, both *T*_1_ and *T*_2_ decrease monotonically (Figure 4c). No clear signature of the structural transition around 200 K is observed, indicating that the spin relaxation process is not sensitive to the change in lattice parameters. At room temperature, *T*_2_ values are on the order of 10 ns. This is significantly lower compared to our previous field-induced ESR measurements on orthorhombic rubrene single crystals,^10^ which found *T*_2_ ∼ 1 μs. This is likely to reflect the absence of counterions in the rubrene lattice, which eliminates a mechanism for spin scattering in the field-induced ESR experiments, and, potentially, the difference in crystal structure (orthorhombic vs triclinic). Nevertheless, for the doped samples, a spin diffusion length of $L_{s}=\sqrt{D\cdot T_{2}}≃210\;\mathrm{nm}$ associated with transport in the vertical direction can be estimated for the 1–5 wt % doped samples (Table 2), with the diffusion coefficient determined by the Einstein relation *D* = *k*_B_ *T*/*e*·μ. Here, we acknowledge that in organic systems with significant energetic disorder, the validity of this relation can be limited, and D is a function of carrier concentration and the width of the density of states. However, given the high crystallinity and steady-state transport conditions of our samples, we assume that the system remains close to equilibrium and that the classical Einstein relation holds for these calculations. These values are of the same order of magnitude as the film thickness of vertical spin transport devices (typically 50–200 nm for spin valves and spin-OLEDs^4,41,42^ ). In the range of 1–5 wt %, the estimated spin diffusion length shows no significant drop with increased doping, which is another beneficial factor for practical spin transport devices because the introduction of the heavily doped top and bottom layers for efficient carrier injection will not affect the spin diffusion process. When the doping level increases to 10 wt %, the spin diffusion length drops to less than 200 nm.

**Table 2:** Extracted Parameters for Carrier and Dopant Concentrations and Extracted Scattering Parameters from ESR Spectra, All Parameters Are at Room Temperature

	1 wt %	3 wt %	5 wt %	10 wt %
density of introduced C_60_F_48_ molecules *n*_C_60_F_48__ (10^18^ cm^–3^)	4.982	14.944	24.908	49.815
spin concentration *n*_spin_ (10^18^ m^3^)	0.831	2.557	4.066	6.244
mobility (cm^2^/(Vs))	1			
spin lifetime *T*_2_ (ns)	18.3	17.6	17.6	16.0
vertical spin diffusion length *L*_s_ (nm)	214.0	210.0	209.8	199.7
effective hyperfine field δ*B*_RMS_^*z*^ (G)	3.81	4.18	4.37	4.45
effective interaction time with dopant τ_1_ (ns)	3.40	4.00	4.61	3.23

The monotonically decreasing *T*_1_ and *T*_2_ with increasing temperature, as shown in Figure 4c, suggest that in the doped rubrene sample, spin relaxation is governed by an inhomogeneous-broadening relaxation process or spin scattering processes that become more efficient as charge carrier motion becomes faster at higher temperatures. We see no evidence of a motional narrowing regime, which would manifest itself as an increase in *T*_2_ with increasing temperature/faster motion. It is possible that at room temperature, where the line width becomes Lorentzian, a motional narrowing regime is being approached. However, it is also possible that such an average effect is due to reduced interaction with the counterion as kinetic energy increases at higher temperatures. The latter argument is supported by the reduced temperature at which the Lorentzian shape becomes dominant in the samples with lower doping levels (Section 5 of the Supporting Information; Figure S11), which is consistent with the trend from our measured activation energy of doping (Table 1).

To describe the spin relaxation processes in this transition regime around room temperature, we applied the modified Redfield theory under the condition γ_e_δ*B*_RMS_^*z*^τ_C_ < 1 ^5,43^

3 $$T_{2}^{\prime }={\left(\frac{1}{T_{2}}-\left(\frac{1}{2T_{1}}\right)\right)}^{-1}=\frac{1}{\gamma_{e}\delta B_{\mathrm{RMS}}^{z}}+\frac{1}{\gamma_{e}^{2}{\left({\delta B}_{\mathrm{RMS}}^{z}\right)}^{2}\tau_{C}}+\tau_{C}$$

Here, τ_C_ is a characteristic lifetime corresponding to the carrier scattering events, γ_e_ is the gyromagnetic ratio of the electron, and δ*B*_RMS_^*z*^ is the width of the Gaussian field distribution of the magnetic field experienced by the carriers, which can be extracted from the measured line width at the lowest temperatures, where carrier motion can be assumed to be completely frozen. We assume in the following that at all temperatures, the spin relaxation is dominated by the interaction with the dopant counterion radical spins; faster motion of the rubrene carriers at higher temperatures leads to more frequent interactions with the dopant spins and a reduction of the spin relaxation time. This is consistent with the clear difference from our previous field-induced EPR studies of spin relaxation in rubrene crystals, in which we saw clear evidence for motional narrowing and longer *T*_2_; the main difference apart from the different polymorph is the absence of spin-carrying counterions in the field-induced ESR experiments. We therefore neglect the 1/γ_e_^2^(δ*B*_RMS_^*z*^)^2^τ_C_ motional narrowing term and extract the effective carrier-dopant interaction time τ_1_ using

4 $$\tau_{1}≃T_{2}^{\prime }-\frac{1}{\gamma_{e}\delta B_{\mathrm{RMS}}^{z}}$$

Table 2 lists the extracted δ*B*_RMS_^*z*^ and τ_1_ for all doping levels at room temperatures, where δ*B*_RMS_^*z*^ are extracted at 10 K, which is the lowest stable temperature at which the line shape fitting can be reliably taken. At 10 K, the extracted τ_1_ is approximately 6 times larger than the disorder-induced relaxation $t_{d}=\frac{1}{\gamma_{e}\delta B_{\mathrm{RMS}}^{z}}$ . Therefore, it is reasonable to assume that the charge motions are frozen out, and hence, δ*B*_RMS_^*z*^ is a good indication of the level of environmental disorder of the material. We noted that the extracted δ*B*_RMS_^*z*^ values, though still in a reasonable range typical for molecular systems, are approximately a factor of 2 smaller than those extracted in our previous report on ion-gel gated rubrene.^10^ This may be attributed to the difference in crystal polymorphs (triclinic vs orthorhombic), or the influence of the interface in the field-induced ESR experiments.

In Table 2, we observe that δ*B*_RMS_^*z*^ increases at higher doping levels, which suggests that the doping subtly influences the local environments experienced by the spin carriers, despite no structural changes being observable in GIWAXS. At room temperature, where the thermal activation results in the greatly reduced τ_1_, τ_1_ shows an increasing trend with increased doping level before dropping at the highest doping level of 10 wt % (Figure 4d inset). This suggests that, except for the highest doping levels, the spin relaxation of the carriers is governed mostly by interactions with individual C_60_F_48_ counterions. As temperature increases, carrier motion in the vicinity of the counterion becomes faster, and the carriers interact more frequently with the counterion, i.e., τ_1_ decreases. As the doping level increases, the carriers become more delocalized and spend a reduced fraction of their time close to the counterion, and τ_1_ increases (or the carriers experience weaker interaction and hence a reduced effective interaction time). At 10 wt % doping, the probability of the carriers interacting with adjacent dopant molecules increases and leads to the observed drop in τ_1_.

From this analysis, we conclude that, to further enhance the spin diffusion length in doped molecular films, it would be interesting to investigate in detail the spin scattering mechanism between the carriers and the dopant counterions. Through careful molecular design of the dopant molecule, it might be possible to minimize the associated spin relaxation processes and achieve longer spin lifetimes.

## 3 Conclusions

We introduced highly doped triclinic rubrene thin-film crystals and analyzed the effectiveness of the dopant C_60_F_48_ by impedance spectroscopy and vertical conductivity measurements. We show that the density of ionized dopants increases almost linearly with the dopant concentration to ∼10^19^ cm^–3^, indicating a high doping efficiency compared to other commonly used dopants such as F_6_-TCNNQ. The temperature-dependent impedance analysis revealed that at dopant concentrations above 3 wt %, the efficiency of the doping process (measured by the activation energy) decreases, which is presumably caused by incomplete ionization or dopant–dopant interaction. The analysis of the vertical conductivity in these films shows that the charge carrier mobility in these films does not depend on the dopant concentration, and it is comparable to the mobility determined by space charge limited currents in undoped films. We provide a conservative estimate of the mobility to be on the order of 1 cm^2^/(V·s). Our ESR analysis showed that the spin susceptibility increases approximately linearly with doping concentration, suggesting that spin pairing effects are not prevalent in this doping regime. We also found that the spin relaxation times of doping-induced carriers in rubrene are much shorter than those of field-induced carriers and are governed by inhomogeneous broadening and interactions with the dopant counterions. Despite this, our study shows that the C_60_F_48_ doped rubrene system is a promising candidate for vertical spintronic devices with sufficiently long spin diffusion length comparable to the film thickness/device dimension. Further advances in spin diffusion length could potentially be made by a better understanding of the molecular structure–property relationships that govern the spin–spin interactions between the rubrene carriers and the dopant counterions.

## 4 Experimental Section/Methods

### 4.1 Sample Fabrication

Devices for impedance and conductivity analysis are built on glass with a size of 150 mm × 150 mm. Substrates are cleaned in acetone, ethanol, isopropanol, and deionized water (dried with nitrogen), followed by a piranha cleaning step.^16^ Layers are deposited via thermal evaporation in a vacuum under a base pressure of 10^–8^ mbar. The evaporation rate of the seed has no influence on the further process. After deposition of the bottom metal electrode and the first amorphous layer of rubrene (40–50 nm), samples are transferred to a nitrogen glovebox, without exposure to air. Heat treatment takes place on a preheated hot plate at 130 °C for 15 min. If needed, additional layers are added using coevaporation of Rubrene and dopant with the same vacuum deposition at rates between 0.5 and 3 Å s^–1^, depending on the doping concentration. Electrodes and the organic layers are structured using shadow masks. Active areas for conductivity and impedance measurements range from 0.04 to 4 mm^2^. The samples are fabricated in a chamber from Kurt J. Lesker Company using a shadow mask system, enabling us to prepare 36 different sample coupons on a 150 mm × 150 mm substrate that can have different thickness or doping concentration. Each coupon hosts four identical devices. Samples for impedance and conductivity analysis are encapsulated in a nitrogen glovebox using a cavity glass and epoxy glue. Samples for ESR are prepared on PFS-5005 UV-fused silica substrates (purchased from UQG Optics) using the same chamber system. Samples for GIWAXS analysis are deposited onto a silicon wafer. Micrographs were taken with a Nikon Eclipse LC100 PL/DS polarization microscope.

### 4.2 Electrical Measurements

Electrical DC measurements are performed using a Keithley 2600 and a Keithley 2635A SMU, and capacitance measurements were performed with a Metrohm Autolab PGSTAT302N potentiostat/galvanostat (Metrohm AG). The impedance spectroscopy is conducted with a root-mean-square amplitude of 20 mV in the potentiostat mode. The measurement is controlled using Nova 2.1.3 software (Metrohm Autolab B.V.) An RC-circuit model is assumed to calculate the capacitance. Impedance measurements dependent on the DC voltage component in reverse direction are performed for each temperature at a frequency of 1 kHz (0.5, 1.0, and 1.5 wt %) or 10 kHz (3 wt %), which is chosen so that the phase is about −90 ° (capacitive behavior) for all temperatures. The bottom and top contacts are gold and aluminum electrodes, respectively. Since the work function of gold is higher than that of aluminum, the latter is expected to form the dominant Schottky contact with the organic. The organic layers are composed of a seed layer (40–50 nm undoped rubrene and rubrene: C_60_F_48_, 5 wt %, 50 nm) and 300 nm rubrene: C_60_F_48_ with various dopant concentrations.

### 4.3 Electron-Spin Resonance

Continuous-wave ESR samples with doped films are measured with a Bruker E500 spectrometer using a Bruker ER 4122SHQE cavity and an X-band microwave source. The modulation frequency is 100 kHz, and the modulation amplitude is 0.2 G above 10 K and 0.3 G below 10 K. The scanned field width is set as 50 G below 50 K, 40 G above 50 K, and centered at 3342 G. About 1024 sampling points of the DC magnetic field are collected from a single spectrum; 3 scans were performed and averaged for each spectrum. The power saturation behavior is measured for microwave power attenuation settings of 50, 40, 30, 25, 20, 16, 12, 10, 8, 6, 4, 2, and 0 dB. The acquisition time is set as 120 s. A tuning of the cavity is performed before scanning, with the Q factor of the cavity between 6000 and 7000 in the presence of the doped sample. The cavity together with the sample is placed in an Oxford Instruments ESR900 cryostat, where the temperature is monitored and controlled by an Oxford Instruments Mercury iTC. The measurements were taken at set temperatures during both warm-up and cool-down cycles to exclude effects from any irreversible degradation effects of the sample during the measurement. The measurements are performed in the following sequence: changing temperature, tuning the cavity, evaluating the Q factor, and taking a measurement. All measurement tools are computer-controlled by the Python code provided in https://github.com/OE-FET/CustomXepr.

### 4.4 Grazing-Incidence Wide-Angle X-ray Scattering

GIWAXS data were obtained at the I07 SAXS/WAXS beamline at the Oxford Diamond Light Source. Two-dimensional scattering patterns were recorded using a Dectris Pilatus 2 M area detector at a beam energy of 20 keV. The sample-to-detector distance was 512.4 mm as determined by a silver behenate reference. Each measurement consists of two 1 s exposures at full beam intensity, with the detector shifted between exposures to fill the module gaps. The samples were placed in a cryostat cooled by liquid nitrogen in the temperature range of 150–290 K for the measurements. Initial data processing was performed using the GIXSGUI MATLAB package, with further analysis in WxDiff.^44^ The coherence length was calculated by using the Scherrer equation

$$L=\frac{2\pi K}{\Delta q_{\mathrm{hkl}}}$$

where *K* is the form factor of the crystal, and Δ*q*_*hkl*_ is the full-width at half-maximum of the Gaussian fit of a specific peak with an arbitrary index (*h*, *k*, *l*) in the reciprocal space. Since we do not have defined diffraction peaks but arcs indicating that the crystallites do not have one preferential orientation to the substrate, we integrated the diffraction signal over the azimuthal angle χ from 10 to 80° (similar to randomly oriented polycrystalline films). We then fitted a Gaussian to the integrated peak. For *K*, we assumed 0.9394 assuming spherical crystallites.^32,45^

### 4.5 Ultraviolet Photoelectron Spectroscopy

Ultraviolet photoelectron spectra of rubrene triclinic (25 nm) on a silicon substrate were measured by using a helium discharge lamp (UVS10/35, Specs) with the He–I excitation line (*h*ν = 21.22 eV) and a Phoibos100 (Specs) measurement system. The base pressure of the vacuum system is 1·10^–10^ mbar, which is increased up to 3·10^–9^ mbar during the measurement. The energy resolution is estimated by measuring the broadening of the Fermi edge of silver with 10 meV. Assuming additional uncertainties due to the evaluation, an inaccuracy in energy of ±50 meV is estimated. The detector is also calibrated by measuring the Fermi level of a sputter-cleaned silver foil.

## Supplementary Material



## Notes

Additional data on the impedance, electrical transport, X-ray diffraction analysis, as well as ESR spectroscopy (PDF)
