# Supplementary material for: Charge and Spin Transport in Doped Rubrene Thin-Film Crystals
Source: ACS Nano. 2026 Mar 16;20(12):10236–45. doi: 10.1021/acsnano.6c02012 (PMC13045350; doi:10.1021/acsnano.6c02012)
Supplement: Supplementary file 1 [file nn6c02012_si_001.pdf]

Supporting Information

Charge and Spin Transport in Doped Rubrene Thin-Film Crystals

*Zichen Wang[A], Stephanie Buchholtz[B], Wooik Jang[B], Mike Hambsch[C], Stefan C. B. Mannsfeld[C], Xinglong Ren[A], Ian E. Jacobs[A], Henning Sirringhaus[A], and Hans Kleemann[B,D]**

[A] Optoelectronics Group, Cavendish Laboratory, University of Cambridge, JJ Thomson Avenue, Cambridge CB3 0HE, United Kingdom

[B] Dresden Integrated Center for Applied Physics and Photonic Materials (IAPP), Nöthnitzer Straße 61, 01069 Dresden, Technische Universität Dresden, Germany
E-mail: hans.kleemann1@tu-dresden.de

[C] Center for Advancing Electronics Dresden and Faculty of Electrical and Computer Engineering, Dresden University of Technology, Dresden, Germany

[D] Nano and Microelectronic System (NMES), Technische Universität Ilmenau, 98693 Ilmenau, Germany
E-mail: hans.kleemann@tu-ilmenau.de

1. Material characterization of the Rub in triclinic phase grown by thermal vapor deposition

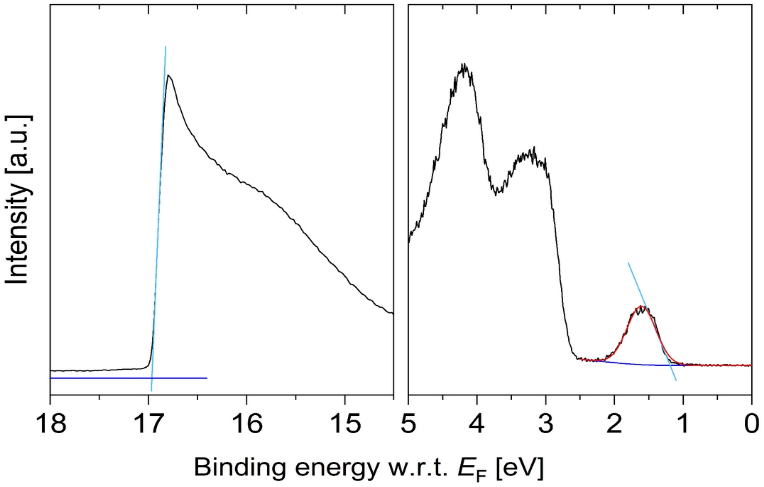

Figure S1: Ultraviolet photoelectron spectroscopy on triclinic rubrene film as prepared for EIS, IV and ESR analysis. Si (substrate) / rubrene triclinic (25 nm). The energies of the high binding energy cut-off E_{HBEC} and the HOMO-onset E_{HOMO} are determined by fitting the peaks with a Gaussian function and calculating the intersection of a tangent through the turning point of the corresponding peak and the background. A Tougaard background was assumed for the HOMO region. The work function WF = $h\nu$ - E_{HBEC} is calculated with (4.65 ± 0.05) eV w.r.t. vacuum and the ionization energy is determined with IE = WF + E_{HOMO} = $h\nu$ - (E_{HBEC} + E_{HOMO}) = (5.43 ± 0.05) eV w.r.t. vacuum.

2. Measured raw data of the Mott-Schottky measurements

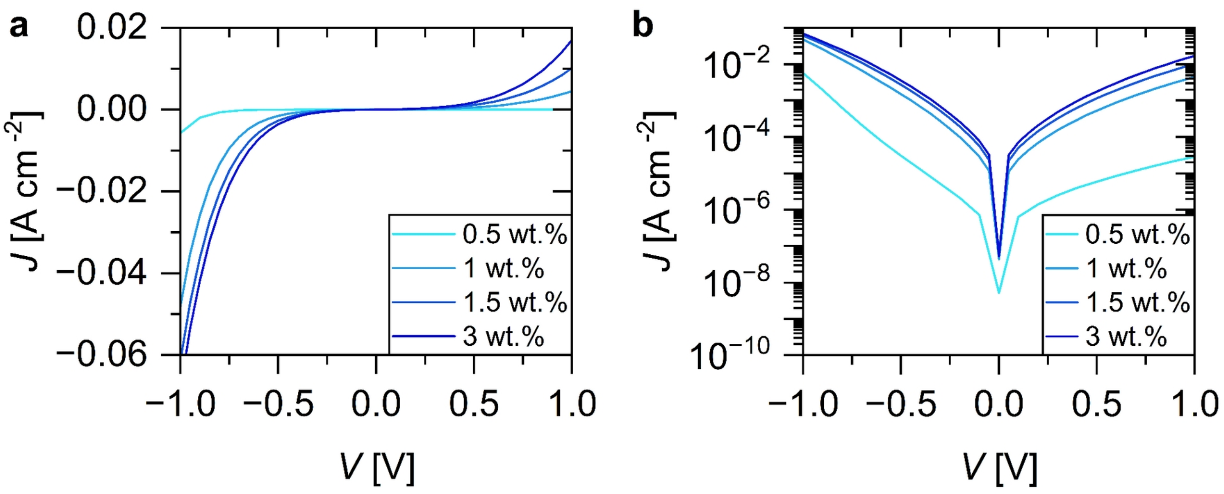

Figure S2. a Linear and **b** logarithmic plotted current density vs. voltage of Schottky diodes with Rub:$C_{60}F_{48}$ for different dopant concentrations with an active area of 0.25 mm^2 measured at room temperature with high potential at top contact (aluminium electrode).

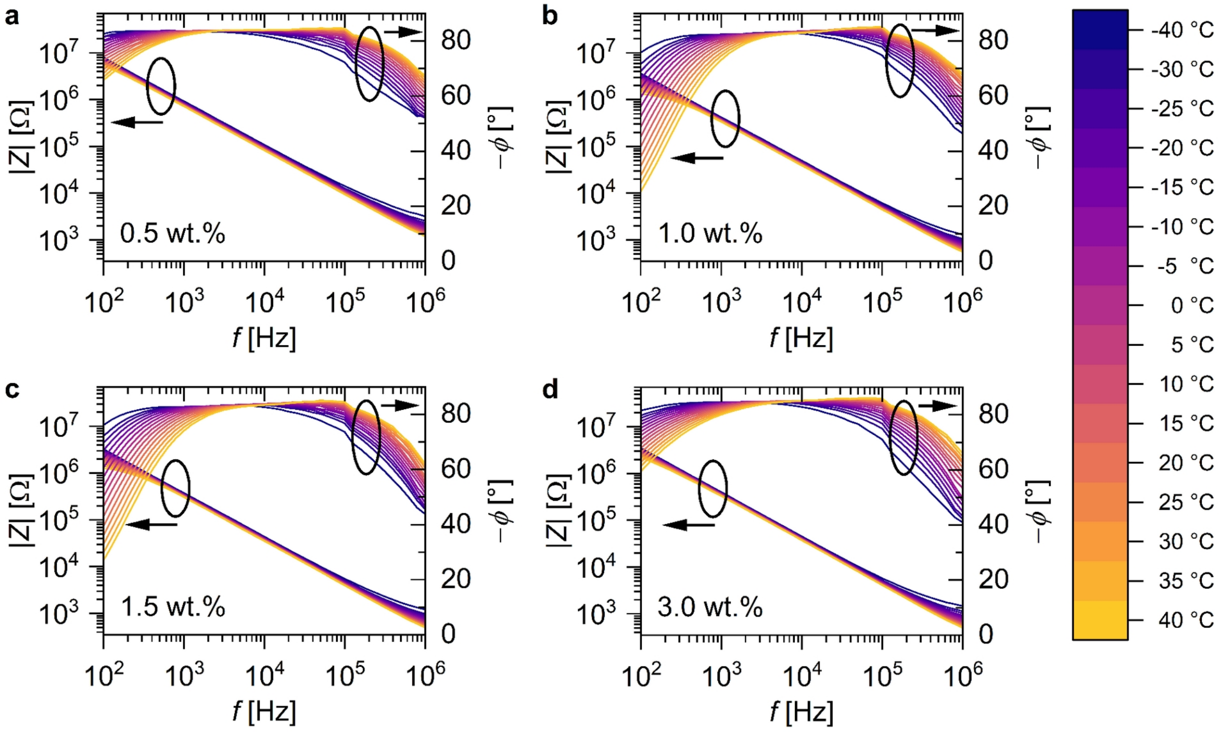

Figure S3. Bode plots for various doping concentrations of rubrene:$C_{60}F_{48}$ at 0 V bias voltage for different temperatures between -40 °C and 40 °C taken at an active area of 0.25 mm². The top contact (aluminum electrode) is at high potential. The maximum of the negative phase - the optimum range for Mott-Schottky analysis - is shifted to lower frequencies for lower temperatures, which indicates a charge carrier freeze-out for low temperatures. The slope of log |Z| (magnitude of impedance) vs. log f (AC frequency) shows a linear and the phase close to -90° over a large frequency range, justifying the analysis using an RC-parallel circuit.

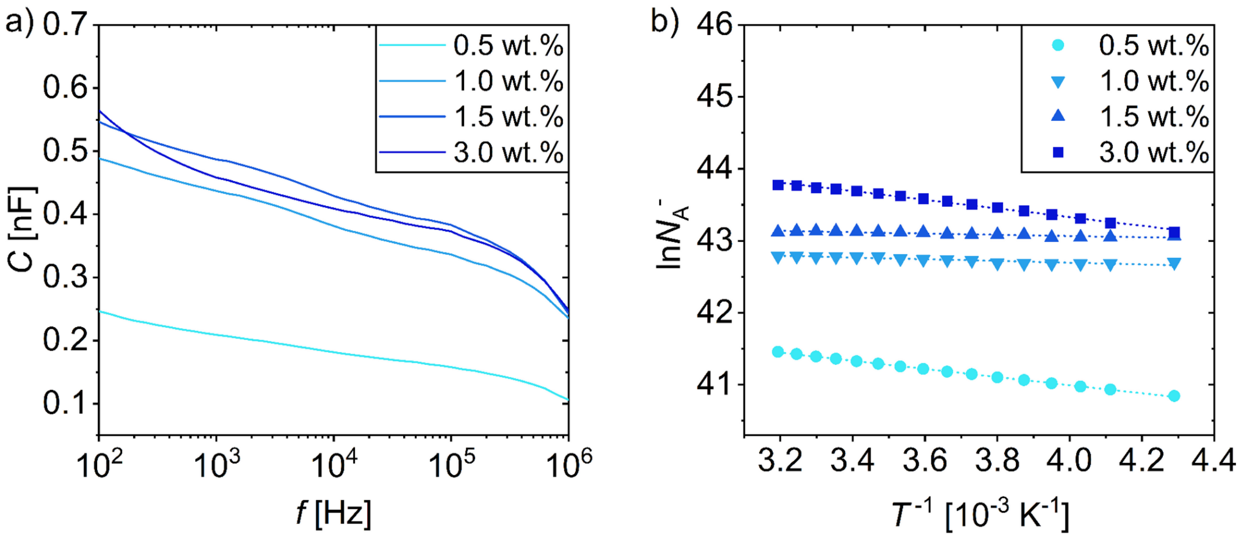

Figure S4. a Capacitance C vs. frequency f at 0 V bias voltage and **b** natural logarithm of the density of ionised acceptor molecules N_A^- (determine from the slope of the Mott-Schottky curve, see Fig.2b) over the inverse of temperature T^{-1} with linear fits for different dopant concentrations, measured at an active area of 0.25 mm^2. The density of ionised acceptors is calculated using Eq.1 and a value of the dielectric constant of rubrene of 2.62 [1].

3. Analysis of the Glazing-incidence wide-angle X-ray scattering data

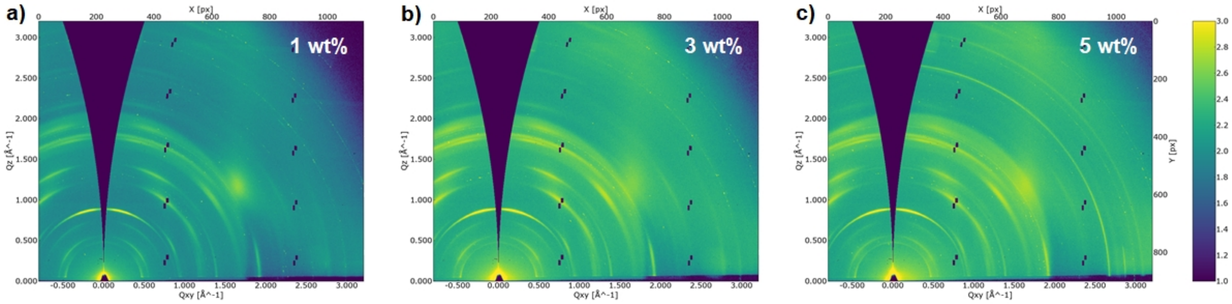

Figure S5: 2D GIWAXS images of rubrene at 295 K for doping concentrations of a) 1 wt.%, b) 3 wt.%, and c) 5 wt.% of $C_{60}F_{48}$.

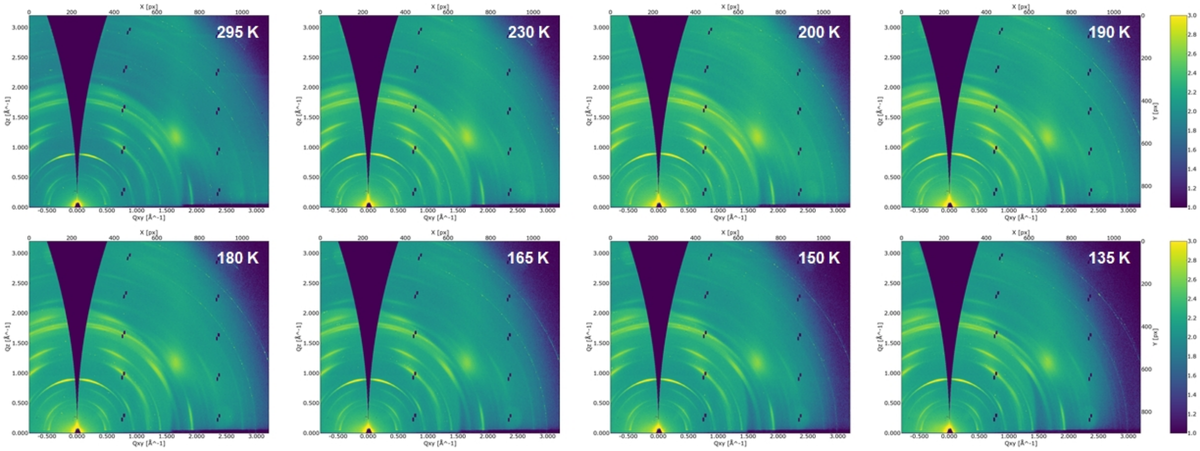

Figure S6. 2D GIWAXS images of rubrene doped with 1 wt.% of $C_{60}F_{48}$ measured at different temperatures.

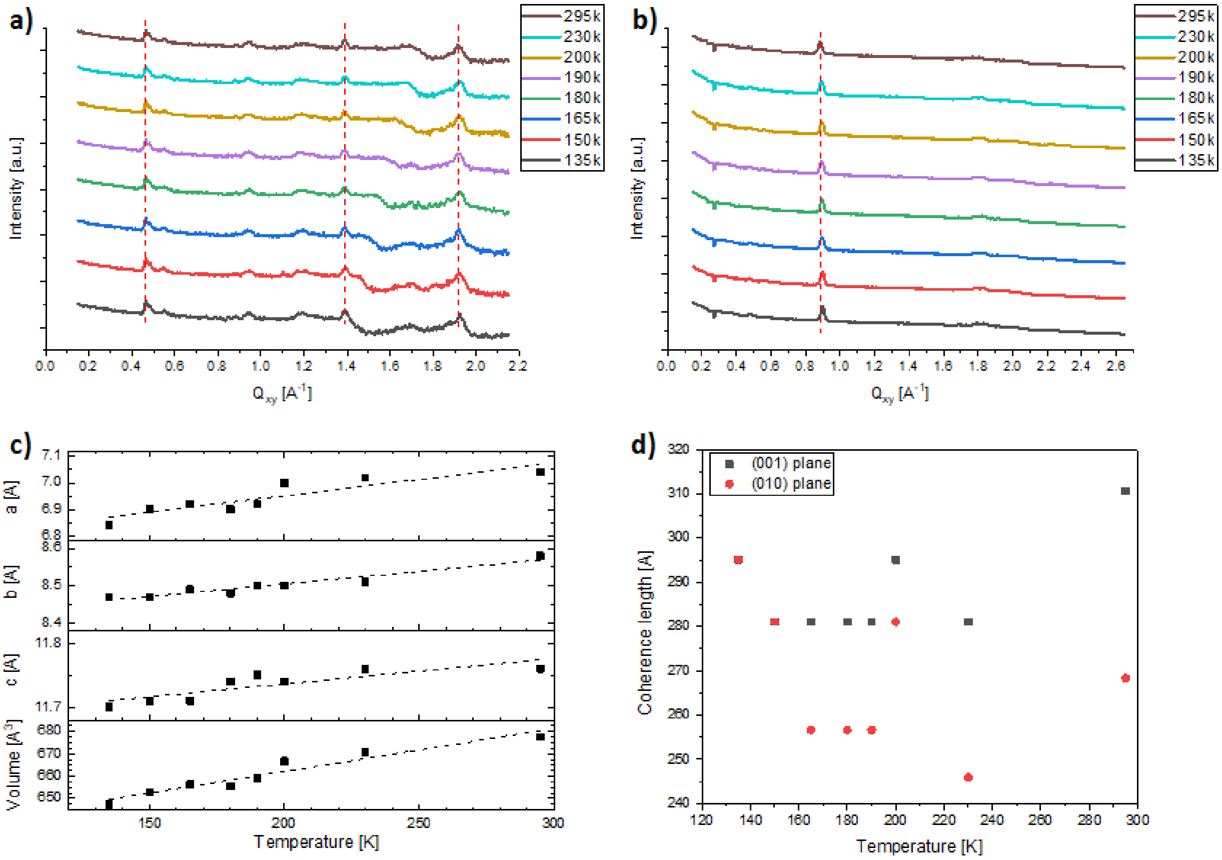

Fig. S7. a) In-plane and b) out-of-plane intensity profiles of the rubrene film doped with 1 wt.% of C60F48 measured at different temperatures. The dashed red lines act as a visual guide for the reader. c) Calculated unit cell parameters and unit cell volume for different temperatures of rubrene doped with 1 wt.% $C_{60}F_{48}$. The unit cell parameters were fitted by assuming that the angles are not changing. d) Calculated coherence length of the (001) and (010) signals.

Doping concentration [wt.%]	0[a]	1	3	5
a [Å]	7.02	7.04	7.06	7.10
b [Å]	8.54	8.58	8.57	8.57
c [Å]	11.95	11.76	11.77	11.84
α [°]	93.04	93.04	93.04	93.04
β [°]	105.58	105.58	105.58	105.58
γ [°]	96.28	96.28	96.28	96.28
Volume [Å^3]	683.4	677.7	679.1	687.4

Table S1. Fitted unit cell parameters of rubrene doped with different concentrations of $C_{60}F_{48}$ measured at 295 K. For the fitting the angles were fixed allowing only for expansion/contraction of the lattice vectors. [a] From literature [2].

Temperature [K]	295	230	200	190	180	165	150	135
**a [Å]**	7.04	7.02	7.00	6.92	6.90	6.92	6.90	6.84
**b [Å]**	8.58	8.51	8.50	8.50	8.48	8.49	8.47	8.47
**c [Å]**	11.76	11.76	11.74	11.75	11.74	11.71	11.71	11.70
**α [°]**	93.04	93.04	93.04	93.04	93.04	93.04	93.04	93.04
**β [°]**	105.58	105.58	105.58	105.58	105.58	105.58	105.58	105.58
**γ [°]**	96.28	96.28	96.28	96.28	96.28	96.28	96.28	96.28
**Volume [Å^3]**	677.7	670.9	666.7	659.1	655.4	656.4	652.9	647.5

Table S2. Fitted unit cell parameters of rubrene doped with 1 wt.% of $C_{60}F_{48}$ measured at different temperatures. For the fitting the angles were fixed allowing only for expansion/contraction of the lattice vectors.

4. Considerations about a signal contribution from $C_{60}F_{48}$ anions

The cw-ESR spectra are dominated by the peak centered at 3342.5 G arising from charge carriers on rubrene. We performed a wide range ESR scan (shown in Figure S8) and found no signature for the $C_{60}F_{48}$ anion, which is expected to be centered at 3346.3 G. According to the Ref [3,4], for C_{60} the ESR signal from the radical anion is observable in both solution and solid state at a g-factor very close to 2. There are two possible explanations for the absence of the signal: 1) $C_{60}F_{48}$ is not stable upon reduction and loses one fluorine forming diamagnetic $C_{60}F_{47}^{-}$ [5]. 2) unpaired electrons are expected to have strong hyperfine interaction with fluorine atoms, which – if unresolved – should largely broadened the signal. Further discussion about the $C_{60}F_{48}$ radical anion species is beyond the scope of this work.

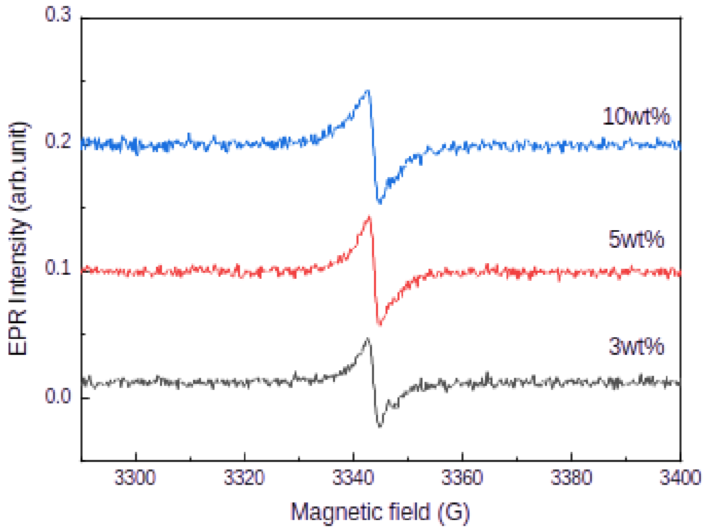

Figure S8 cw-ESR spectra recorded at various doping levels with broad window of magnetic field scanning. Measurement is done at room temperatures, with the applied microwave power of 2mW.

5. Process to analyze the ESR spectra

For the quantitative study of ESR data, we used a 2-dimensional power saturation fitting function defined as [6]

$$S_{A,T_1,T_2,\delta B_{rms},B_0}(B,B_{mw}) = A \cdot \frac{B_{mw}}{\Gamma(B_{mw})} \cdot \frac{\partial}{\partial B} Voigt\left[B - B_0; \frac{2}{\gamma_e T_2}\Gamma(B_{mw}), \delta B_{rms}\right] \quad \text{(S1)}$$

, where the input variable B is the applied DC magnetic field for each scan of ESR spectrum, and B_{mw} are the AC magnetic field in the centre of the ESR cavity generated by the microwave power. Following our previous work, this value can be extracted from the applied microwave power P_{mw} and the effective cavity Q-factor

$$B_{mw} = 2.4069\left(T \cdot W^{-1/2}\right)\sqrt{P_{mw} \cdot Q} \quad \text{(S2)}$$

The power saturation factor Γ is expressed as

$$\Gamma(B_{mw}) = \sqrt{1 + \gamma_e^2 T_1 T_2 B_{mw}^2} \quad \text{(S3)}$$

$Voigt[x; \Delta x_L, \Delta x_G]$ is the Voigt function defined as the convolution of a Gaussian (with standard deviation Δx_G) and Lorentzian function (with full width at half maximum Δx_L)

$$Voigt[x; \Delta x_L, \Delta x_G] = \int_{-\infty}^{+\infty} G(x'; \Delta x_G) L(x - x'; \Delta x_L) dx' \quad \text{(S4)}$$

so that both the spin lifetime T_2 and the inhomogeneous broadening of the spectrum are taken into account (the Gaussian function $G(x'; \Delta x_G)$ and Lorentzian function $L(x'; \Delta x_L)$ are scaled to have a unit area after integration). The parameters A is the scaling factor of the ($A \cdot B_{mw}$ is the peak area, i.e. twice integration of the measured spectra at each B_{mw}); $T_1$is spin-lattice relaxation time; T_2 is spin-spin relaxation time, δB_{rms} is the standard deviation corresponding to the Gaussian lineshape as a measure of the magnetic field inhomogeneity; B_0 is the centre of the peaks. The extracted scaling factor is then converted into the susceptibility (in unit of m^3) as

$$\chi = k \cdot A \cdot \frac{\mu_0 g_e^2 \mu_B^2}{3\hbar \omega_{mw} B_{mod}} \quad \text{(S5)}$$

, where ω_{mw} is the angular frequency of the applied microwave power, g_e is the effect g-factor determined by the peak centre from $\hbar\omega_{mw} = g_e \mu_e B_0$, B_{mod} is the modulation field for the continuous wave lock-in detection; μ_0, μ_B $\hbar$ are the vacuum permeability, Bohr magneton, and the reduced Planck constant, respectively. k is a conversion coefficient obtained by calibration to the standard BDPA sample (supplied by Bruker, Series Ref: ER-213-BDS) and then a series of TEMPO (2,2,6,6-Tetramethylpiperidine 1-oxyl, 2,2,6,6-Tetramethyl-1-piperidinyloxy) radical samples with a series of concentrations (200, 400, 600 μM). The start pointing for the 2D fitting

of A, T_2, δB_{rms}, and B_0 are chosen from the 1D fitting of $A \cdot Voigt\left[B - B_0; \frac{2}{\gamma_e T_2}, \delta B_{rms}\right]$ function to the spectrum with maximum amplitudes, while the T_1 value is determined by fitting of $A \cdot \frac{B_{mw}}{\Gamma(B_{mw})}$ function to the twice integration of the spectra with all B_{mw} values. Following the steps described above, we are able to determine the χ, T_1, T_2 and δB_{rms} simultaneously. An example of the fitting is shown in Figure S8.

(a) (b)

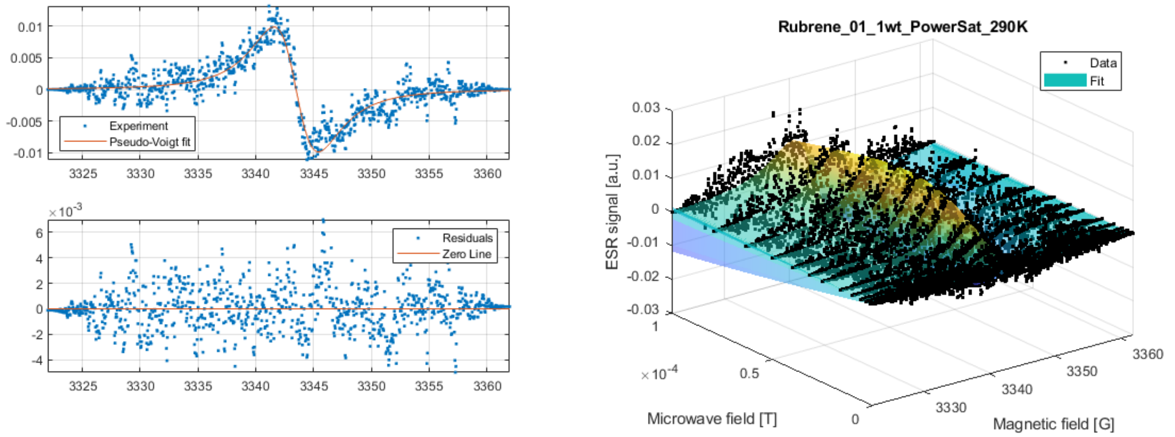

Figure S9 An example of 2D fitting of 1wt% doping levels at 290 K, (a) The initial fitting of the spectrum with the intermediate microwave power, (b) the original data and the fitting of the 2D curve

In order to verify that carriers are transported in the inhomogeneous broadening regime, we evaluate the residuals of the fitting and compare the fitting results using the Voigt function with the Lorentzian function. In the inhomogeneous broadening regime, the inhomogeneity in the molecular environment of carriers is reflected in the measured spectra, which is believed to have a Gaussian distribution in the energy scale. Otherwise, if the peak width is purely limited by the intrinsic lifetime in an exponential decaying factor in the time domain, the spectra are expected to have a Lorentzian shape.

Figure S10 shows the change of residuals, the Voigt function is preferable in all temperature ranges, while the Lorentzian function shows a comparable fitting result only at room temperature. A fitting example of which two types of functions show comparable results is shown in Figure S9. It can be seen that the Voigt function overestimated the peak width at the intermediate MW power, whereas the Lorentzian function is narrower than the measured data at the edges of the peaks. We argue that the Voigt function still fits better the measured curve in this case. The broadened peak width is likely due to the imprecise estimation of the linewidth broadening in Voigt function as the power saturation term enters the Lorentzian components only in Equ (S1).

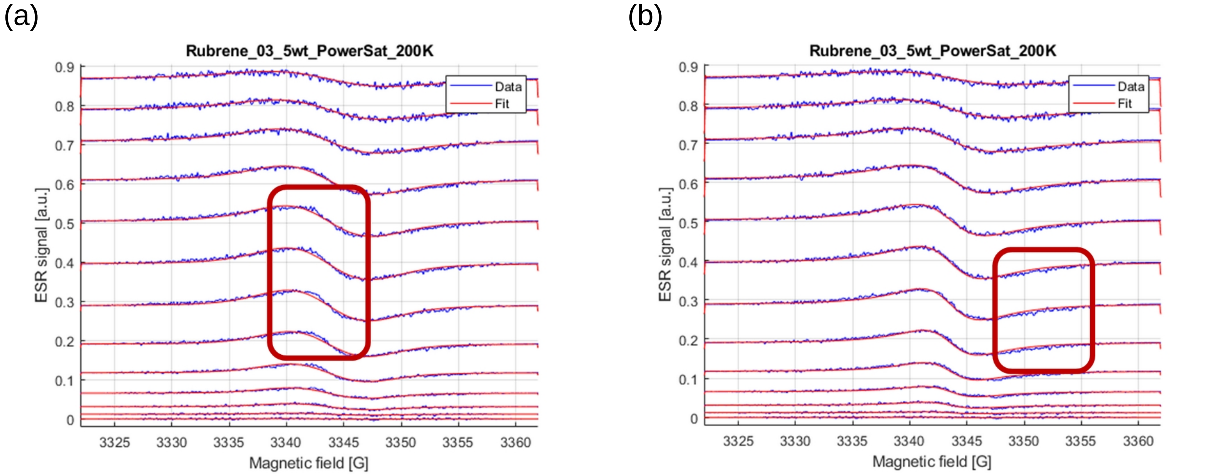

Figure S10. The comparison of the 2D fittings with the measured data points using (a) Voigt and (b) Lorentzian functions at 5wt% dopants and 200K, stacked curves in each plots shows the spectra recorded at different microwave power (from bottom to top is the increased power following the sequence listed in the Methods)

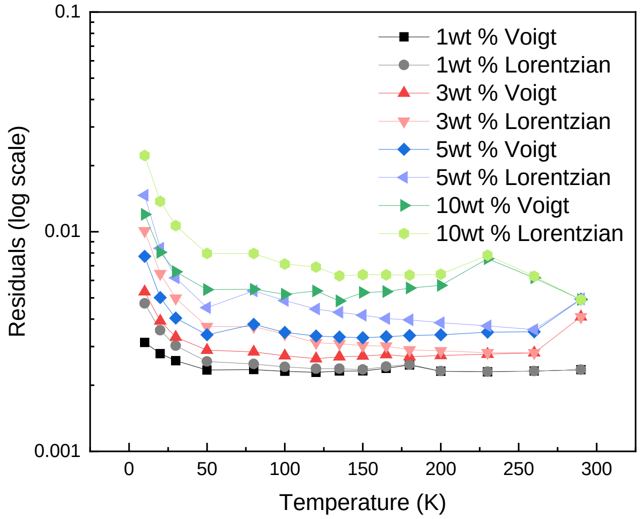

Figure S11. the residuals of the 2D fittings using Voigt and Lorentzian functions at various temperatures and doping levels

6. Susceptibility and carrier concentrations for the cw-ESR analysis

For the isolated spin which follows Curie's law, the susceptibility follows the equation

$$\chi_C = \frac{\mu_0 \mu_B^2}{3 k_B T} g^2 S(S+1) N_{spin} = \frac{\mu_0 \mu_B^2}{k_B T} N_{spin} \quad \text{(S6)}$$

, where μ_B is Bohr magneton; μ_0 is the vacuum permeability, and k_B is the Boltzmann constant, N_{spin} is the total number of spin carrier, which can be converted to the carrier density n_{spin} by division of the volume of the sample. The carrier concentration n_{spin} extracted from the χ values at 290K are listed in Table S3, and compared with the carrier density extracted from MS and expected dopant concentration n_{dopant} set by the depositing conditions:

$$n_{dopant} = \frac{1}{v_0} \times \frac{M_{Rub}}{M_{C60F48}} \times wt\,\% \quad \text{(S7)}$$

, where the volume of a unit cell at 290K (from the GIWAXS data) is $v_0 = 655\,Å^3 = 6.55 \times 10^{-28} m^3$; the rubrene molar mass is $M_{Rub} = 532.7\, g/mol$, and the $C_{60}F_{48}$ molar mass is $M_{C60F48} = 1632.6\, g/mol$.

Table S3 Carrier concentrations extracted from cw-ESR and charge transport measurements, compared with doping concentrations from the fabrication steps, units for the concentration is $10^{18} cm^{-3}$

	n_{spin} from cw-ESR	$N_A^{-¿¿}$ from MS	n_{dopant} from doping level
1wt%	0.831	3.79	4.982
3wt%	2.557	9.48	14.944
5wt%	4.066	-	24.908
10wt%	6.244	-	49.815

n_{spin} from cw-ESR shows comparable values with the number of free carriers extracted from MS data. Apart from spin pairing effects these differences could also be accounted for by these measurements not being performed on the same samples/device architecture. Based on the observed g-factor g_0 = 2.0022 extracted from the peak centre, which is close to the g_0 in intrinsic rubrene crystals[7], we argue that ESR peaks correspond to the mobile rubrene carriers induced by doping. There could also be errors in the ESR estimates of the carrier concentration associated with the large sample area used for the ESR devices needed to induce a sufficient number of spins. This requires a high uniformity of the doped films. As seen from the GIWAXS patterns, there films comprise a mixture of the triclinic phase with the orthorhombic phase, that could affect the ESR estimate of carrier concentration. A more refined estimation of carrier concentration is beyond the scope of this paper. However, we believe the ESR estimate carrier densities are broadly consistent with the MS results.

References

[1] S. J. Konezny, M. N. Bussac, L. Zuppiroli, *Appl Phys Lett* **2009**, *95*, 263311.
[2] T. R. Fielitz, R. J. Holmes, *Cryst Growth Des* **2016**, *16*, 4720.
[3] J. Niklas, K. L. Mardis, O. G. Poluektov, *J Phys Chem Lett* **2018**, *9*, 3915.
[4] V. I. Krinichnyi, E. I. Yudanova, N. G. Spitsina, *Journal of Physical Chemistry C* **2010**, *114*, 16756.
[5] A. V. Belyakov, R. Y. Kulishenko, R. D. Johnson, I. F. Shishkov, A. N. Rykov, V. Y. Markov, V. E. Khinevich, A. A. Goryunkov, *J Phys Chem A* **2020**, *124*, 10216.
[6] S. Schott, U. Chopra, V. Lemaur, A. Melnyk, Y. Olivier, R. Di Pietro, I. Romanov, R. L. Carey, X. Jiao, C. Jellett, M. Little, A. Marks, C. R. McNeill, I. McCulloch, E. R. McNellis, D. Andrienko, D. Beljonne, J. Sinova, H. Sirringhaus, *Nat Phys* **2019**, *15*, 814.
[7] Y. Takahashi, M. Tsuji, Y. Yomogida, T. Takenobu, Y. Iwasa, K. Marumoto, *Applied Physics Express* **2013**, *6*, 041603.
